# Prevalence of Target Organ Damage in Chinese Hypertensive Children and Adolescents

**DOI:** 10.3389/fped.2018.00333

**Published:** 2018-11-19

**Authors:** Liu Yang, Lili Yang, Yuanyuan Zhang, Bo Xi

**Affiliations:** Department of Epidemiology, School of Public Health, Shandong University, Jinan, China

**Keywords:** children and adolescents, hypertension, target organ damage, prevalence, China

## Abstract

**Background:** Subclinical target organ damage (TOD) has been common in hypertensive children, but there is limited data in the Chinese pediatric population. This study aimed to investigate the prevalence of subclinical TOD in the Chinese hypertensive children and adolescents.

**Methods:** A cross-sectional study was performed in children and adolescents from four schools in Jinan, China between September 2012 and September 2014. The hypertensive status was confirmed based on elevated blood pressure across three different occasions. Those with hypertension were invited to participate in the evaluation of TOD (including heart, arteries, and kidney) and metabolic disorders. A total of 7,840 children and adolescents aged 6–17 years were recruited at baseline, of whom 373 were diagnosed as hypertensive after three separate visits, and 333 (89%) participated in evaluation of TOD.

**Results:** Among 333 hypertensive children, 47.4% had elevated carotid intima-media thickness, 32.4% had left ventricular hypertrophy, 29.2% had dyslipidemia, 7.6% had liver dysfunction, and 4.1% had microalbuminuria. Cardiovascular damages were more prevalent in children aged 9–14 years than the other age groups (i.e., 6–8 and 15–17 years). Girls had higher proportion of microalbuminuria than boys (8.2 vs. 2.6%). No significant sex and age differences were observed for the prevalence of other TODs. Nearly all subclinical TODs were much more prevalent in hypertensive children who were overweight and obese than those with normal weight, except microalbuminuria.

**Conclusions:** The subclinical TOD is prevalent in Chinese hypertensive children and adolescents. Effective measures should be taken to fight against subclinical TOD.

## Introduction

With the obesity epidemic among youths, pediatric hypertension has been an alarming public health issue worldwide. Hypertension in childhood can find its way into adulthood (1) and adulthood hypertension is the main cause of the global cardiovascular disease burden, leading to 10.7 million deaths and 211.8 million disability-adjusted life-years in 2015 (2). Recent evidence suggests that pediatric hypertension is also associated with the risk of subclinical target organ damage (TOD) in childhood (3). Subclinical TOD manifests as injuries to several target organs, including the heart, arteries, and kidneys (3), which are risk factors for future cardiovascular events. The Bogalusa Heart Study clearly showed that the major etiologies of adult cardiovascular disease originated from childhood (4). The US Fourth Report recommended that screened hypertensive children should undergo TOD assessment and treatment, if necessary in clinical practice, and the presence of subclinical TOD is regarded as a sign for pharmacologic treatments to control hypertension (5). On the other hand, if hypertension is controlled adequately, at least some of the subclinical TOD can be reversible (6).

To our knowledge, there is limited data on the prevalence of subclinical TOD in Chinese hypertensive children. Thus, in the present study, we targeted children and adolescents from the general population, who were screened as hypertensive after three separate visits, to report the prevalence of subclinical TOD among hypertensive children and adolescents in Jinan, China.

## Materials and methods

### Study population

This study was performed from September 2012 to September 2014 in Jinan, China. Four public middle-level schools (2 primary schools, 1 junior high school, and 1 senior high school) in the urban region of Jinan were selected using convenient cluster sampling method. The sampling unit was the school. In the first stage, all participants in the selected schools were invited to fill in standard questionnaires (e.g., demographic information) and underwent physical examination including measurements of weight, height, and blood pressure (BP). A total of 7,840 school children aged 6–17 years (boys: 4,082, girls: 3,758) with complete data were included for data analysis. In the next stage, 333 of 373 school children (40 subjects were lost to follow-up) who were diagnosed with hypertension after repeated BP measurements on three separate occasions participated for further evaluation of subclinical TOD. The study was approved by the Ethics Committee of the Capital Institute of Pediatrics in Beijing, China and written informed consent was obtained from all the school children and their parents or guardians.

### Physical examination

Anthropometric data were obtained by trained research staff based on a standardized protocol at the initial visit. Body weight was measured twice using a standard scale (at 0.1 kg) for school children in light clothing without shoes, and height was also measured twice, to the nearest 0.1 cm. The mean values were used for data analysis. Body mass index (BMI) was calculated as the ratio between weight and the square of height (kg/m^2^). Being overweight and obesity were defined using the sex- and age-specific BMI percentile cut-offs (7).

BP was measured with an appropriate cuff size on the right arm at the heart level, in a seated position. All measurements were performed with the electronic sphygmomanometer (OMRON HEM-7012), which was validated for children and adolescents (8). Three BP readings were obtained and the last two values were averaged for data analysis. Elevated BP in children and adolescents was defined as systolic and/or diastolic BP equal to or above 95th percentile values by sex and age, according to references for Chinese children's and adolescents' BP (9). Children with elevated BP at the first visit underwent the second visit after 2 weeks, using the same procedures. If elevated BP persisted during the second visit, those children were required to undergo BP measurement at the third visit 2 weeks later. Subjects whose BP levels remained elevated after the three separate occasions were defined to be hypertensive according to the pediatric BP guideline (5, 10).

### Heart assessment

Sonographers used Doppler ultrasonography device (Sonos 5500, Andover, MA, USA) with a 2.5–4.0 MHz probe to assess the left ventricular structure and function of the subjects. Left ventricular end-diastolic internal dimension, interventricular septal thickness, and posterior wall thickness were measured. Left ventricular mass (LVM) was calculated using Devereux's formula LVM (g) = 0.8 × [1.04 × (left ventricular end-diastolic internal dimension + interventricular septal thickness + posterior wall thickness)^3^ − (left ventricular end-diastolic internal dimension)^3^] + 0.6 (11). Left ventricular mass index (LVMI) was calculated as LVM (in grams) / height ^2.7^ (in meters) to correct for body size (12). Left ventricular hypertrophy (LVH) was defined as LVMI ≥ age- and sex-specific 95th percentile values; a single-partition value of 51 g/m^2.7^ was the cut-off for severe LVH (10, 13, 14). The relative wall thickness (RWT) was calculated as (posterior wall thickness + interventricular septal thickness) / left ventricular end-diastolic internal dimension and the cut-off point of 0.42 was used to define concentric geometry (10). Based on LVMI and RWT, the children were then divided into four groups including normal geometry (normal LVMI and RWT), concentric remodeling (normal LVMI but increased RWT), eccentric hypertrophy (increased LVMI but normal RWT), and concentric hypertrophy (increased LVMI and RWT) (10).

### Vascular assessment

Carotid intima-media thickness (cIMT), an index of early vascular damage, was measured by sonographers using the Doppler ultrasonography device paired with a 5–10 MHz probe. The wall of the carotid artery was clearly presented. The cIMT was measured at the common carotid artery, along a 10 mm section, proximal to the common carotid sinus. We measured four cIMT values on both the right and left sides, and the mean value was calculated for data analysis. High cIMT was defined using the sex- and age- specific 95th percentile values (15).

### Renal assessment

Early renal damage was defined by the presence of microalbuminuria. Mid-stream urine samples were collected from each participant after fasting >12h overnight to evaluate the albumin, uric acid, and urinary creatinine concentrations. Microalbuminuria was defined as the ratio of albumin and creatinine being between 3 and 30 mg/mmol (13). Albumin < 3 mg/mmol creatinine was considered normal.

### Liver assessment

Aspartate aminotransferases (AST) and alanine aminotransferases (ALT) were assessed using the Hitachi 7080 Chemistry Analyzer. ALT or AST > 40 U/L was considered to be abnormal (16).

### Lipid and glucose assessments

After fasting >12h overnight, venous blood samples were collected in tubes containing EDTA. Centrifugation was performed at 3,000 rpm for 10 min to separate the plasma and cells. The plasma samples were then freeze-stored and shipped to the central laboratory of the Capital Institute of Pediatrics in Beijing for further testing, including levels of serum total cholesterol, triglycerides, low-density lipoprotein cholesterol, high-density lipoprotein cholesterol and fasting plasma glucose measured using the Hitachi 7080 Chemistry Analyzer. Pediatric dyslipidemia was diagnosed as the presence of at least one of the following: total cholesterol ≥ 5.18 mmol/L, triglycerides ≥ 1.13 mmol/L for those aged 6–9 years or triglycerides ≥ 1.47 mmol/L for those aged 10–17 years, high-density lipoprotein cholesterol ≤ 1.04 mmol/L, and low-density lipoprotein cholesterol ≥ 3.37 mmol/L (17). Impaired fasting plasma glucose was defined as fasting plasma glucose ≥ 5.6 mmol/L (18).

### Statistical analysis

Data were analyzed using SAS version 9.3 (SAS, Cary, North Carolina, USA). Quantitative variables were presented as means ± standard deviations (or standard errors), and the categorical variables were expressed as percentages. To adjust for age and sex, z scores for growth indicators (i.e., height, weight, BMI, and BP) were calculated, with the reference data from China Health and Nutrition Survey (19). Differences in mean values of TOD indicators between groups were compared using covariance analysis adjusted for age and sex. Chi-square test was used for categorical variables. Two-sided *p* < 0.05 was considered to be statistically significant.

## Results

A total of 333 school children (boys: 71.5%), who were hypertensive after three separate visits, finally participated in the assessment of TOD. Boys had higher height and weight and systolic BP, and were more likely to be obese, but they had lower diastolic BP than girls (*p* < 0.05). Similar results were presented by z scores of these indicators controlling for age and sex, with the reference data from China Health and Nutrition Survey (Table 1). Moreover, the prevalence of obesity was high in each age group, with the corresponding figures as 71.7, 62.9, 74.2, and 52.9%, in age groups of 6–8, 9–11, 12–14, and 15–17 years, respectively.

**Table 1:** Characteristics of children who participated in the evaluation of target organ damage.

	**All**	**Boys**	**Girls**	***p-*value**
N	333	238	95	
Age (years)	12.71 ± 3.18	12.86 ± 3.07	12.32 ± 3.42	0.157
Height (cm)	159.56 ± 16.92	162.30 ± 16.95	152.60 ± 14.80	< 0.001
Height-z score	1.17 ± 0.85	1.27 ± 0.83	0.94 ± 0.87	0.002
Weight (kg)	68.42 ± 23.78	72.39 ± 23.66	58.46 ± 21.11	< 0.001
Weight-z score	2.67 ± 1.74	2.80 ± 1.73	2.35 ± 1.71	0.032
Body mass index (kg/m^2^)	26.04 ± 5.62	26.73 ± 5.39	24.30 ± 5.85	< 0.001
Body mass index-z score	2.27 ± 1.71	2.34 ± 1.70	2.10 ± 1.75	0.240
Systolic blood pressure (mmHg)	132.96 ± 10.86	135.20 ± 10.80	127.20 ± 8.68	< 0.001
Systolic blood pressure-z score	2.68 ± 0.75	2.73 ± 0.77	2.56 ± 0.68	0.084
Diastolic blood pressure (mmHg)	71.85 ± 8.02	71.16 ± 8.24	73.64 ± 7.16	0.012
Diastolic blood pressure-z score	0.52 ± 1.27	0.33 ± 1.36	1.01 ± 0.82	< 0.001
Overweight (%)	21.0	21.4	20.0	0.030
Obesity (%)	64.0	66.8	56.8	

*Data are shown as mean ± standard deviations or percentages*.

*To make the data comparable, z scores for each indicator adjusted for age and sex are calculated with the China Health and Nutrition Survey as the reference data*.

Levels of cIMT were significantly higher in boys than in girls, while other markers were not significantly different between boys and girls. Levels of RWT, cIMT, uric acid, urinary creatinine, ALT, and AST were significantly different within age groups; however, there were no discrepancies regarding metabolic data (Table 2).

**Table 2:** Mean values of target organ damage indices and biochemical indicators by sex and age.

**Target organ damage indices**	**All (*N* = 333)**	**Sex**			**Age group**				
		**Boys**	**Girls**	***p*-value**	**6–8**	**9–11**	**12–14**	**15–17**	***p-*value**
		**(*N* = 238)**	**(*N* = 95)**		**(*N* = 53)**	**(*N* = 62)**	**(*N* = 97)**	**(*N* = 121)**	
**HEART**									
Left ventricular mass index (g/m^2.7^)	36.8 ± 0.7	37.1 ± 0.9	36.0 ± 1.4	0.503	37.9 ± 1.8	35.7 ± 1.7	37.9 ± 1.3	36.0 ± 1.2	0.604
Relative wall thickness	0.36 ± 0.01	0.36 ± 0.01	0.36 ± 0.01	0.899	0.35 ± 0.01	0.39 ± 0.01	0.37 ± 0.01	0.33 ± 0.01	< 0.001
**VESSEL**									
Carotid intima-media thickness (mm)	0.46 ± 0.01	0.47 ± 0.01	0.44 ± 0.01	< 0.001	0.42 ± 0.01	0.43 ± 0.01	0.48 ± 0.01	0.47 ± 0.01	< 0.001
**KIDNEY**									
Uric acid (mmol/L)	371.1 ± 6.2	370.2 ± 7.2	373.3 ± 11.6	0.821	327.9 ± 14.3	372.3 ± 13.0	393.6 ± 10.3	369.8 ± 11.8	0.004
Urinary creatinine (mmol/L)	11.9 ± 0.3	12.2 ± 0.4	11.2 ± 0.6	0.147	9.6 ± 0.7	11.9 ± 0.7	13.1 ± 0.6	12.1 ± 0.5	0.002
Albumin (mg/L)	9.7 ± 1.8	9.3 ± 2.1	10.8 ± 3.4	0.698	7.4 ± 4.3	7.8 ± 4.0	8.6 ± 3.3	12.8 ± 3.0	0.640
**LIVER**									
Alanine aminotransferases (U/L)	15.6 ± 0.8	16.5 ± 1.0	13.1 ± 1.6	0.067	13.7 ± 2.0	11.9 ± 1.8	18.4 ± 1.4	16.1 ± 1.6	0.031
Aspartate aminotransferases (U/L)	27.5 ± 0.5	27.9 ± 0.6	26.5 ± 0.9	0.209	30.8 ± 1.2	29.1 ± 1.1	27.4 ± 0.8	24.1 ± 1.0	< 0.001
**METABOLIC INDICATORS**									
Total cholesterol (mmol/L)	3.7 ± 0.1	3.7 ± 0.1	3.8 ± 0.1	0.425	3.9 ± 0.1	3.6 ± 0.1	3.7 ± 0.1	3.8 ± 0.1	0.276
Triglycerides (mmol/L)	1.0 ± 0.1	1.0 ± 0.1	0.9 ± 0.1	0.063	1.0 ± 0.1	0.9 ± 0.1	1.1 ± 0.1	1.0 ± 0.1	0.159
High-density lipoprotein (mmol/L)	1.3 ± 0.1	1.3 ± 0.1	1.4 ± 0.1	0.054	1.4 ± 0.1	1.3 ± 0.1	1.3 ± 0.1	1.3 ± 0.1	0.097
Low-density lipoprotein (mmol/L)	2.1 ± 0.1	2.1 ± 0.1	2.1 ± 0.1	0.850	2.2 ± 0.1	2.1 ± 0.1	2.1 ± 0.1	2.1 ± 0.1	0.793
Fasting plasma glucose (mmol/L)	5.4 ± 0.1	5.5 ± 0.1	5.3 ± 0.1	0.158	5.3 ± 0.1	5.4 ± 0.1	5.6 ± 0.1	5.4 ± 0.1	0.119

*Data are shown as mean± standard errors*.

*Covariance analyses adjusted for age and sex are performed to assess the differences in each TOD indices across sex or age groups*.

**Figure 1:**
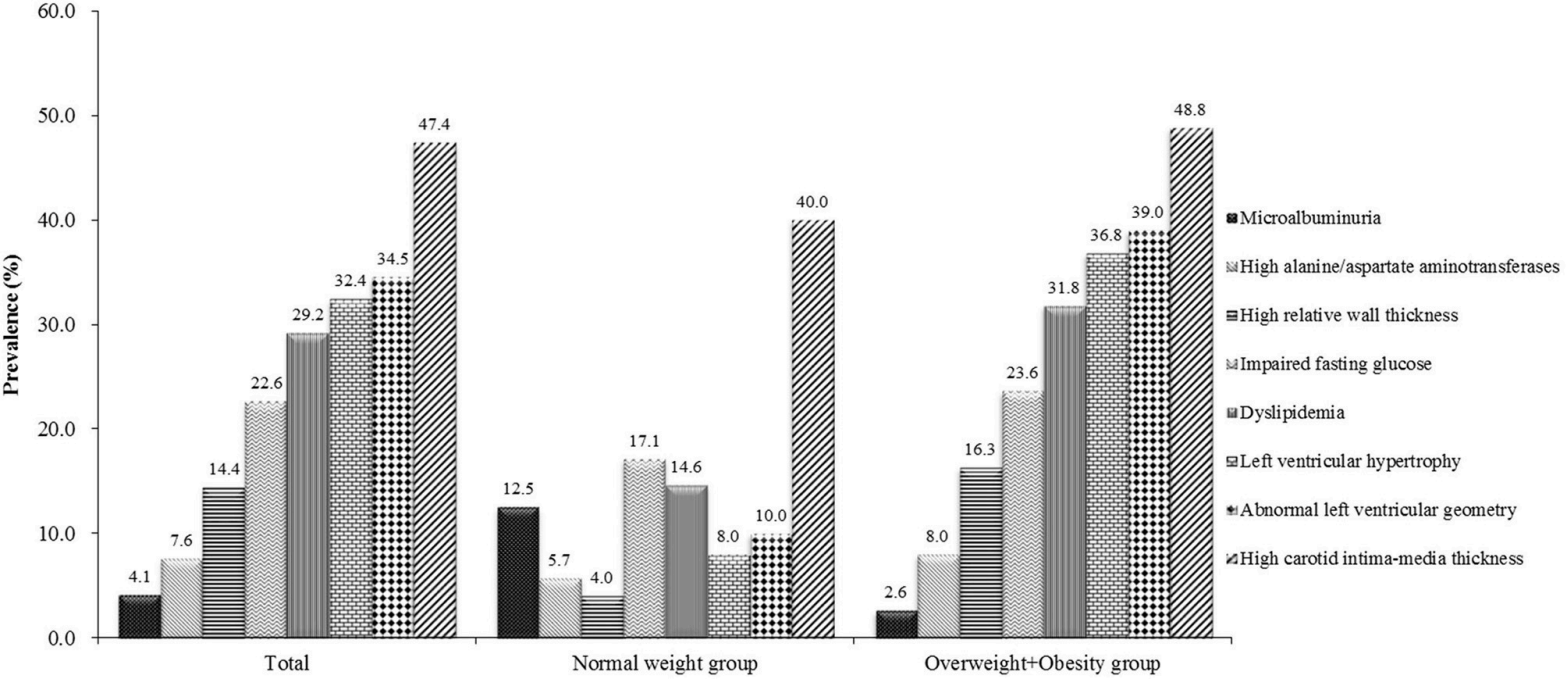
Prevalence of target organ damage in Chinese hypertensive children and adolescents by weight status.

Among 333 school children with hypertension, 47.4% had elevated cIMT, 34.5% had abnormal left ventricular geometry (LVG) (including 12.2% who had concentric hypertrophy, 20.2% who had eccentric hypertrophy, and 2.1% who had concentric remodeling), 32.4% had LVH (15.0% with severe LVH), 29.2% had dyslipidemia and 22.6% had abnormal fasting plasma glucose, 7.6% had abnormal liver function, and 4.1% had microalbuminuria. The prevalence of subclinical TOD was much higher in hypertensive children who were overweight and obese than those with normal weight, except microalbuminuria (Figure 1). There was significant difference in the prevalence of abnormal LVG and elevated cIMT across age groups, with the prevalence being higher in children aged 9–14 years. Girls had a higher proportion of microalbuminuria than boys (8.2 vs. 2.6%). No sex difference was observed regarding the prevalence of other TOD and metabolic abnormalities (Table 3).

**Table 3:** Prevalence of target organ damage and metabolic abnormalities by sex and age.

	**Sex**			**Age group (years)**				
	**Boys**	**Girls**	***p*-value**	**6–8**	**9–11**	**12–14**	**15–17**	***p*-value**
	**(*N* = 238)**	**(*N* = 95)**		**(*N* = 53)**	**(*N* = 62)**	**(*N* = 97)**	**(*N* = 121)**	
**HEART**								
Left ventricular hypertrophy	32.2	33.0	0.895	28.3	40.3	34.7	28.2	0.340
High relative wall thickness	14.0	15.4	0.746	22.6	30.7	15.8	0.9	< 0.001
Abnormal left ventricular geometry								
Concentric hypertrophy	11.4	14.3	0.748	22.6	29.0	9.5	0.9	< 0.001
Eccentric hypertrophy	20.8	18.7		5.7	11.3	25.3	27.4	
Concentric remodeling	2.5	1.1		0.0	1.6	6.3	0.0	
**VESSEL**								
High carotid intima-media thickness	51.0	38.6	0.056	32.4	36.8	61.5	43.8	0.005
**KIDNEY**								
Microalbuminuria	2.6	8.2	0.049	3.8	3.3	3.3	5.5	0.848
**LIVER**								
High alanine aminotransferases	5.9	4.6	1.000	2.4	0.0	11.1	4.8	0.034
High aspartate aminotransferases	3.5	6.1	0.472	7.3	2.0	4.9	3.2	0.597
High alanine aminotransferases or aspartate aminotransferases	7.7	7.6	0.985	7.3	2.0	12.4	6.4	0.169
**METABOLIC ABNORMALITIES**								
High total cholesterol	1.1	1.3	1.000	4.1	0.0	1.2	0.0	0.151
High triglycerides	18.2	14.5	0.469	30.6	10.0	17.1	13.9	0.030
Low high-density lipoprotein cholesterol	19.3	11.7	0.137	14.3	16.7	20.5	15.3	0.772
High low-density lipoprotein cholesterol	1.1	1.3	1.000	4.0	0.0	1.2	0.0	0.158
Dyslipidemia	31.6	23.4	0.184	40.8	25.0	30.1	23.6	0.184
Impaired fasting glucose	23.7	20.0	0.523	12.2	21.7	25.6	27.1	0.232

*Data are shown as percentages*.

*Chi-squared analyses are performed to assess the differences in prevalence of TOD across sex or age groups*.

## Discussion

The present study showed that subclinical TOD was common in Chinese hypertensive children and adolescents. The prevalence of elevated cIMT and LVH was 47.4 and 32.4%, respectively. In addition, cardiovascular damages were more prevalent in children aged 9–14 years.

LVH is a well-established independent risk factor for cardiovascular morbidity and mortality in adulthood. In this study, we used two criteria to define LVH. One was based on the age- and sex-specific 95th percentile values of LVMI (pediatric criteria) proposed by Khoury et al. (14). The other one was based on the cutoff of 51 g/m^2.7^ (adult criteria) recommended by the 2017 updated American Academy of Pediatrics guideline (10), which had been shown to predict risk of cardiovascular disease in adults. Using the criteria proposed by Khoury et al. (14), one study conducted in Michigan showed that 35% (49 of 141) of hypertensive children had LVH (20). In addition, Litwin et al. reported that the prevalence of LVH was 46.5% in 86 children and adolescents with untreated primary hypertension (6). Based on the adult criteria (LVMI ≥ 51g/m^2.7^), one previous study of 129 children with hypertension showed that the prevalence of severe LVH was 15.5% (21). Despite possible ethnic difference in LVM levels, the prevalence of LVH seemed to be similar, especially for severe LVH. All these studies demonstrated that LVH was prevalent in hypertensive children. However, only a quarter of children with hypertension underwent echocardiography examination to assess the presence of LVH (22), though it was recommended as a routine examination in pediatric hypertensive patients (5, 10). LVH can be further described by LVG, classified by the presence of LVH or high RWT (10), which was also associated with cardiovascular disease outcomes in adults. A cross-sectional study of 141 hypertensive children reported that 40% had abnormal LVG, including 11% who had concentric hypertrophy and 16% who had eccentric hypertrophy (20), which are similar to our study.

cIMT, the most common method to assess vascular structure, has been regarded as an index of increased cardiovascular risks. A study reported that children with hypertension had elevated cIMT compared to their control groups (0.46 ± 0.06 vs. 0.35 ± 0.12 mm) (23). Kollias et al. reported that cIMT was correlated with systolic BP (24). These results are consistent with our findings. Furthermore, in our study, cardiovascular damages were more prevalent in children aged 9–14 years old. This may be due to intense hormonal changes and elevated insulin resistance during this early pubertal period. Actually, evidence has suggested that puberty exerts negative influence on endothelial function and antioxidant mechanisms (25).

Kidney is another target organ impaired in hypertensive children. In 2012, Seeman et al. reported that the incidence of microalbuminuria was 20% in children with hypertension (26), much higher than our result (4.1%). Different methods used for urine collection and the severity of hypertension may explain the discrepancy between the two studies. The study by Seeman et al. used random urine samples, while our study used morning urine samples. Evidence suggested that morning urine samples usually contain lower albuminuria than daytime samples due to daily physical activities.

In the present study, the prevalence of obesity among hypertensive children and adolescents was high (64.0%). Based on data from the first occasion (*n* = 7,840), the prevalence of obesity was 20% in the general population. In addition, the prevalence of obesity was also low (17.5%) in normal BP children from the same population. This is expected since obesity is a well-documented main risk factor for childhood hypertension. Previous studies also showed that the prevalence of overweight and obesity ranged from 51 to 72% among hypertensive children, which are consistent with our findings (20, 27). The prevalence of most TOD was much higher in overweight and obese children than their normal weight counterparts in our study, except for microalbuminuria. This finding is as expected since overweight and obesity are well-documented risk factors of TOD (28). However, microalbuminuria was more prevalent in normal weight hypertensive children. This can be explained by the fact that normal weight children usually do more physical activity than obese ones, and hence they may be more likely to excrete more albumin (29). Additionally, we found that the prevalence of an increased cIMT was high in both normal weight and obese children although it was more prevalent in obese children, which indicates that childhood hypertension may be associated with vascular damage independent of the effect of obesity. Our finding is consistent with previous studies (30).

The current study showed comprehensive descriptions of hypertension-related TOD including the heart, arteries, kidney damages, and dyslipidemia in hypertensive children and adolescents screened across three separate visits from the general population. However, our study also has several limitations. First, only children who were diagnosed with hypertension were assessed TOD measures, and there were no normal BP controls in our study. Thus, we cannot assess whether hypertensive children are more likely to develop related TOD. Future studies should consider using age-, gender-, and ethnicity-matched healthy controls for the comparison. Second, we used Chinese children and adolescents' BP references (9) to define elevated BP across three visits, which made our results incomparable with other studies. Third, BP re-assessments were only made in children who had elevated readings on the preceding visits. This may cause some underestimation in prevalence of hypertension, especially when some children had normal BP at initial visit, but actually had elevated BP at subsequent visits. Fourth, there was less consensus on criteria used for the definitions of TOD in the pediatric population, and some criteria were based on the adult references, which may lead to under-estimations for the true abnormal status. Fifth, it is worth mentioning, however, that the reproducibility of TOD measurements was not assessed in the present study, which may impact the stability of results. Sixth, other more sensitive indices of subclinical target organ damage, e.g., arterial stiffness measurements, cardiac strain imaging echocardiography, and liver transient elastography were not assessed in the present study.

## Conclusions

In conclusion, our study demonstrates that subclinical TOD in hypertensive children and adolescents is prevalent. Efforts should be made to fight against hypertension and subclinical TOD in children, which include non-pharmacologic interventions (e.g., adoption of healthy lifestyles) and pharmacologic treatments. These treatments will be effective in reversing the presence of TOD in children and adolescents, as proved by previous studies (6).

## Author contributions

LY and BX conceptualized the study and drafted the article. LY, LLY, and YZ analyzed data. LY, LLY, and BX also made contributions to revising the manuscript. All authors approved the final submitted and published versions.

### Conflict of interest statement

The authors declare that the research was conducted in the absence of any commercial or financial relationships that could be construed as a potential conflict of interest.
